# Prognostic Evaluation for Patients over 45 Years Old with Gallbladder Adenocarcinoma Resection: A SEER-Based Nomogram Analysis

**DOI:** 10.1155/2020/6370946

**Published:** 2020-07-18

**Authors:** Pengfei Li, Lujun Song

**Affiliations:** Department of General Surgery, Zhongshan Hospital, Fudan University, Shanghai 200032, China

## Abstract

Gallbladder adenocarcinoma is the main histopathological type of gallbladder cancer (GBC), so it is particularly important to understand its biological characteristics. Due to the low incidence of this type of cancer, there are few studies with large sample sizes. The log of positive lymph nodes (LODDS) has been evaluated by many scholars as a lymph node stage that may play a better role than the 8^th^ edition of the American Joint Committee on Cancer (AJCC) lymph node staging system in many cancers. However, the effect of LODDS has not been proven in gallbladder adenocarcinoma. Our research aimed to identify independent prognostic factors that are closely related to overall survival (OS) in patients with gallbladder adenocarcinoma over 45 years of age using data from the Surveillance, Epidemiology and, End Results (SEER) database. All patients were randomly divided into a modeling cohort and an internal validation cohort. Seven independent prognostic factors associated with OS—age, marital status, grade, tumor size, AJCC 8^th^ edition T stage and M stage, and LODDS—were used to build a nomogram to predict 1-, 3-, and 5-year survival. The C-index of our nomogram was 0.735 (95% CI, 0.716 to 0.754), and together with the calibration curve and ROC curve validation, the results confirmed the prediction effect of our nomogram. We believe that our nomogram will be an accurate and convenient method for patient prognosis assessment in the future.

## 1. Introduction

Gallbladder cancer (GBC) is the most common tumor of the biliary tract with the worst overall survival [1]. This is because GBC is usually diagnosed when the tumor has progressed and is already large enough to block nearby structures [2]; therefore, the overall survival (OS) rate of GBC is still low: the 5-year OS rate of unresectable GBC is no more than 5% [3–5]. At the very beginning, most patients only have gallstones in their gallbladder [2]. If not handled promptly, chronic inflammation caused by gallstones can stimulate the gallbladder mucosa and eventually lead to GBC [2, 5]. However, radical surgery can significantly improve patient prognosis regardless of the tumor stage. Therefore, the early identification and management of patients with a good prognosis is very important.

Gallbladder adenocarcinoma is the main type of GBC, accounting for approximately 80% [6, 7]. According to data from the Surveillance, Epidemiology, and End Results (SEER) database, 97.3% of patients with GBC are middle-aged (older than 45), so the evaluation of good prognosis in this patient group is urgent. Although many studies have reported some prognostic factors for GBC, including age, TNM stage, tumor size, adjuvant therapy, and pathological grade, there has not yet been a population-based study that has systematically clarified postoperative patients over 45 years of age [6, 8, 9]. The 8^th^ edition of the AJCC TNM staging system has just been put into clinical use, so it is urgent to establish a prediction model based on this staging system to assist in daily clinical use [10]. The log of positive lymph nodes (LODDS) has been evaluated by many scholars as a lymph node stage that may play a better role than the 8^th^ edition of the AJCC lymph node staging system [11, 12]. A better prognosis prediction model could be established by combining these two different evaluation models. The aim of our study is to use the SEER database to assess the independent prognostic factors for patients who underwent gallbladder adenocarcinoma resection and were older than 45 years old and to provide individualized guidance for the surgical prognosis of this population.

## 2. Materials and Methods

### 2.1. Patient Selection

**Figure 1:**
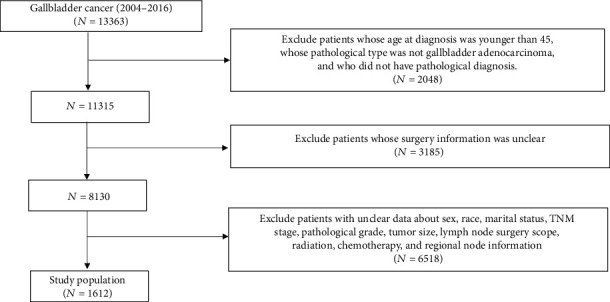
The study flow diagram of the selection process.

We extracted the clinical data of patients with GBC from 2004 to 2016 from the SEER database, which covers up 28% of the US population and 97% of tumor categories. The exclusion criteria we used were as follows: (1) patients whose pathological type was not gallbladder adenocarcinoma or who did not have a pathological diagnosis; (2) patients whose age at diagnosis was younger than 45 years old; (3) patients whose surgery information was unclear or patients without surgery; and (4) patients with unclear data regarding sex, race, marital status, TNM stage, pathological grade, tumor size, lymph node surgery scope, radiation, chemotherapy, or regional node examination information. Our data selection method is shown in Figure 1 and Table S1. The selected data were divided into a training cohort and a validation cohort by a ratio of approximately 3 to 1, the same as in previous similar studies [13, 14]. Data downloaded from the SEER database did not require patients' informed consent and may be reproduced or copied without permission.

In our research, we used the variables of sex, race, marital status, age at diagnosis, AJCC TNM stage, pathological grade, lymph node surgery scope, tumor size, radiation, and chemotherapy. The 8^th^ AJCC TNM staging system was used to identify the stage of gallbladder adenocarcinoma [10]. The variable RX Sum (1998+) was used to extract tumor size. The tumor's histologic subtype was identified by specific coding data embedded in the SEER database; i.e., code 8140 represented adenocarcinoma. LODDS was calculated by using the following formula: log [(0.5 + the amounts of positive LNs)/(0.5 + the amounts of negative LNs)] [11]. The LODDS value in our cohort ranged from -4.39 to 3.30. We used X-tile software to obtain the best cut-off values for LODDS and tumor size (Figure S1, 15). LODDS was divided into LODDS1 (-4.39 to -1.47), LODDS2 (-1.47 to 0.90), and LODDS3 (0.90 to 3.30), and tumor size was grouped into <1.7 cm, 1.7-3.3 cm, and ≥3.4 cm.

### 2.2. Statistical Analysis

SPSS 20.0 statistical software (SPSS Inc., Chicago, IL, USA) and R software version 3.6.1 (The R Foundation for Statistical Computing, Vienna, Austria. http://www.r-project.org) were used to run statistical analysis. The Pearson chi-squared test was used to describe the clinical characteristics. Univariate and multivariate analyses were conducted by the Cox proportional hazards regress1ion model, and hazard ratios (HRs) together with the corresponding 95% confidence intervals (CIs) were calculated. Variables with *P* < 0.05 in univariate analysis were incorporated into multivariate analysis. The Kaplan-Meier method was used to estimate OS, and different survival curves were analyzed by log-rank tests. *P* values were calculated by two sides, and *P* < 0.05 was considered statistically significant for all of our analyses.

The rms package from R was used to formulate a nomogram based on the independent prognostic factors from multivariate analysis. The concordance index (C-index), Akaike information criterion (AIC), ROC curve, and calibration plots were used to evaluate the nomogram as described previously [14]. The C-index denoted a low accuracy between 0.50 and 0.70, a medium accuracy between 0.71 and 0.90, and a high accuracy over 0.90. The calibration curve was a scatter plot of the actual and predicted incidence values in order to compare the predicted OS and the observed OS.

## 3. Results

### 3.1. Clinicopathologic Features of Patients

This present study identified 1612 patients with gallbladder adenocarcinoma between 2004 and 2016; 1212 patients were in the training cohort, and 400 patients were in the validation cohort. The clinicopathologic features of the patients in the training cohort and validation cohort are presented in Table 1. There was no statistically significant difference between the two groups for all data. The majority of patients in both cohorts were relatively old (≥65 years old), female, white and married. The most common tumor grade was moderately differentiated (775, 48.1%), followed by poorly differentiated (596, 37.0%). According to the 8^th^ AJCC TNM staging system, the primary T-stage was T2 (802, 49.8%), and the primary M-stage was M0 (1365, 84.7%). The majority of patients who underwent removal of 1-3 regional lymph nodes accounted for 67.0%. Only 678 (42.1%) patients underwent chemotherapy, and 385 (23.9%) patients underwent radiation. There were 895 (55.5%) patients with a tumor size of 1.9–4.8 cm, 355 (22.0%) patients with a tumor size of <1.9 cm, and 362 (22.5%) patients with a tumor size of ≥4.8 cm. LODDS was divided into LODDS1 (-4.39 to -1.47), LODDS2 (-1.47 to 0.90), and LODDS3 (0.90 to 3.30) by X-tile software. The medium-risk cohort (LODDS2) consisted of 642 (39.8%) patients, followed by LODDS1 (518, 32.1%) and LODDS2 (452, 28.0%).

### 3.2. Prognostic OS Analysis by Univariate and Multivariate Cox Analyses

In general, the median follow-up time was 15 months (range, 0–154 months), and the median OS was 23 months (95% CI: 20.52–25.47 months). The 1-, 3-, and 5-year OS rates for all patients were 68.3%, 37.3%, and 28.3%, respectively. Through univariate testing for the training cohort, factors including age, marital status, race, grade, tumor size, T stage, LODDS, and M stage showed statistical significance with OS (*P* < 0.05). In addition, sex, LN surgery scope, radiation, and chemotherapy failed to reach statistical significance (*P* > 0.05) (Table 2).

**Figure 2:**
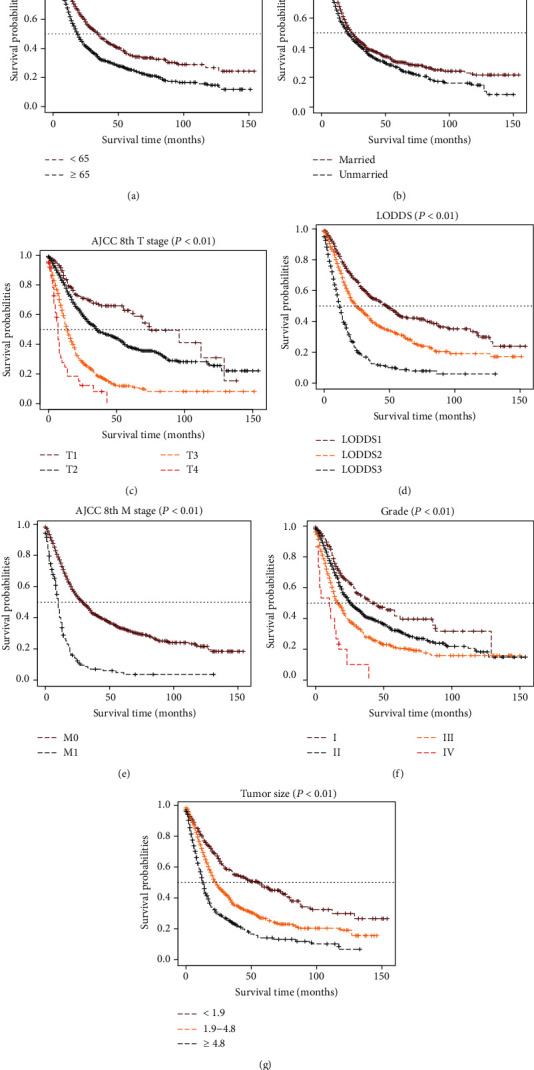
KM OS curves stratified by independent prognostic factors: (a) age, (b) marital status, (c) AJCC 8th T stage, (d) LODDS, (e) AJCC 8th M stage, (f) tumor grade, and (g) tumor size. AJCC: American Joint Committee on Cancer; LODDS: log of positive lymph nodes.

Seven independent survival predictors associated with OS, age, marital status, grade, T stage, M stage, tumor size, and LODDS were selected by multivariate Cox regression analysis (Table 2). A patient who was unmarried (HR = 1.298, 95% CI: 1.117-1.508, *P* < 0.001) and older than 65 years (HR = 1.686, 95% CI: 1.4430-1.988, *P* < 0.001) and who had poorly differentiated pathological grade IV (HR = 2.295, 95% CI: 0.898–5.066, *P* = 0.005), T4 stage (HR = 3.715, 95% CI: 2.262-6.101, *P* < 0.001), and M1 stage (HR = 2.067, 95% CI: 1.704-2.508, *P* < 0.001) disease as well as a tumor size larger than 4.8 cm (HR =1.503, 95% CI: 1.179–1.916, *P* < 0.001) was considered to have a worse prognosis. In particular, patients with LODDS2 (HR = 1.316, 95% CI: 1.087–1.593, *P* = 0.005) or LODDS3 (HR = 2.515, 95% CI: 2.053–3.080, *P* < 0.001) appeared to have a worse prognosis than those with LODDS1. Through Kaplan-Meier survival analysis, we concluded that the above independent factors of prognosis had statistical significance for OS in their respective strata (Figure 2).

### 3.3. Prognostic Nomograms for OS

**Figure 3:**
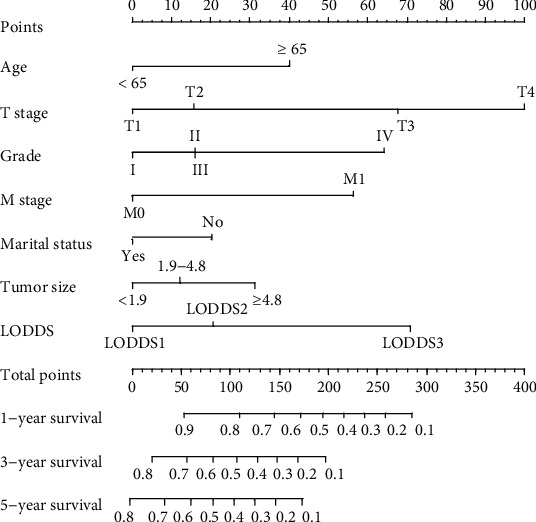
Nomogram to predict 1-, 3-, and 5-year overall survivals in patients who underwent gallbladder adenocarcinoma resection. LODDS: log of positive lymph nodes.

**Figure 4:**
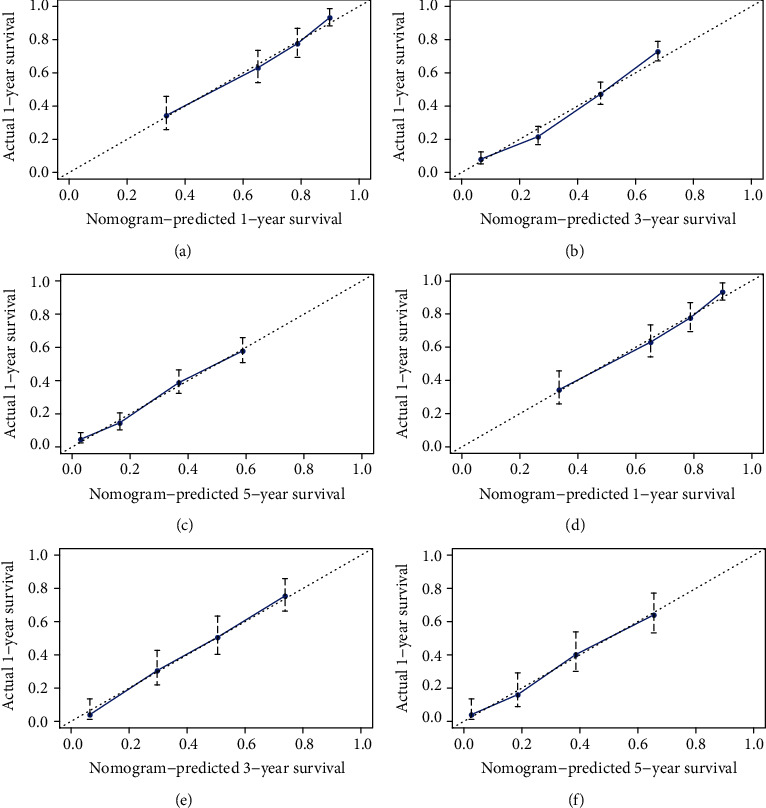
1-, 3-, and 5-year OS forecasted nomogram calibration graphs for the training cohort (a-c) and internal validation cohort (d–f). The *x*-axis represents the OS probability predicted by the nomogram; the *y*-axis represents the actual survival rate. Diagonal lines represent ideal reference column diagrams. Striped dots represent the probability of the nomogram prediction and the 95% confidence interval.

**Figure 5:**
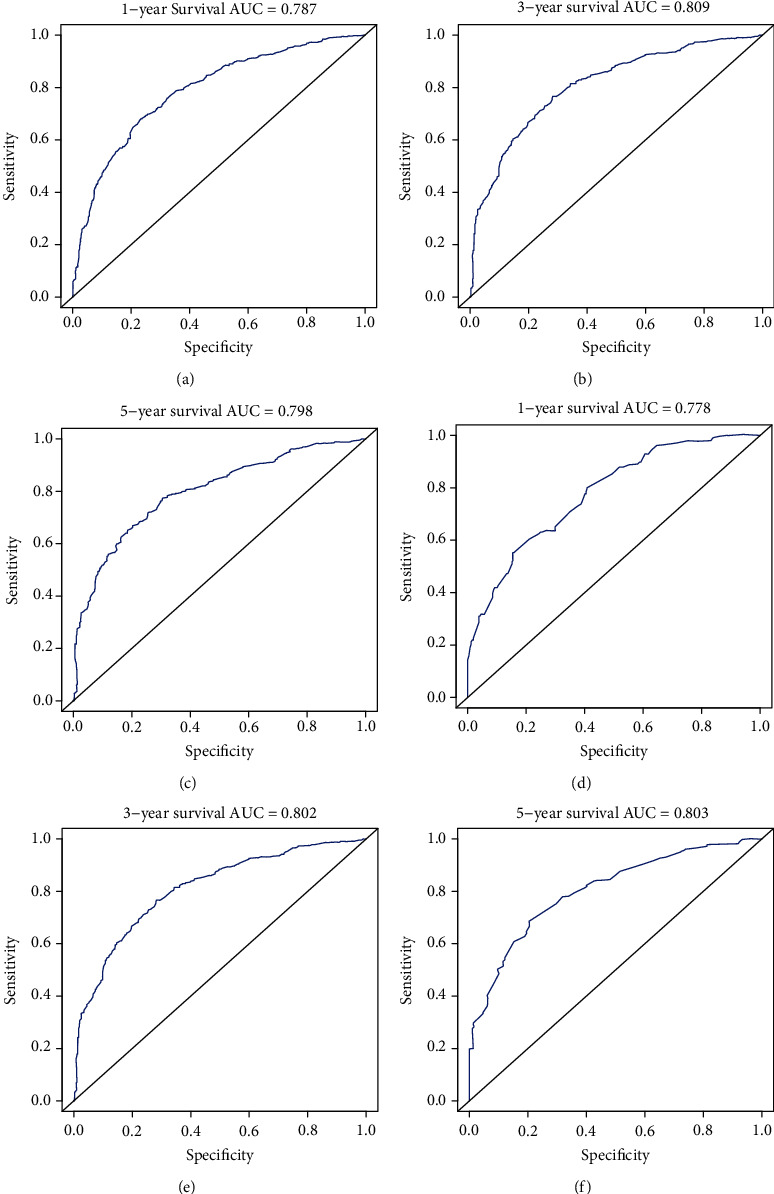
AUC value of the ROC predicting approximately 1-, 3-, and 5-year OS forecasted nomograms for the training cohort (a–b) and internal validation cohort (d–f).

The prognostic nomogram was constructed by the independent survival predictors obtained from the results of the multivariate analysis described above (Figure 3). The C-index for OS prediction in the training cohort and validation cohort was 0.735 (95% CI, 0.716 to 0.754) and 0.740 (95% CI, 0.721 to 0.759). The C-index of the 8^th^ edition of the AJCC TNM staging system in the training cohort and validation cohort was 0.698 (95% CI: 0.678-0.717) and 0.693 (95% CI: 0.674-0.712). The AIC of our nomogram in the training cohort and validation cohort was 8658 and 2324. The AIC of the 8^th^ edition of the AJCC TNM staging system in the training cohort and validation cohort was 8763 and 2375. Our nomogram shows a better discrimination and prediction ability than the 8^th^ edition of the AJCC TNM staging system. The calibration curve and ROC curve for the survival rate in the training cohort and validation cohort showed good consistency between prediction and observation at one, three, and five years (Figures 4 and 5.

## 4. Discussion

In our research, we used the data from the SEER database to set up a nomogram for OS in approximately 1612 patients over 45 years old who underwent gallbladder adenocarcinoma resection. Although many established nomograms of GBC consider radiotherapy, chemoradiotherapy, and adenocarcinoma using the SEER database [6, 9, 15, 16], there is no GBC population-based research regarding specific pathology types or age groups based on LODDS.

Our study is the first population-based study using the SEER database to establish a prognostic nomogram for patients older than 45 who underwent gallbladder adenocarcinoma resection based on the 8^th^ edition of the AJCC TNM staging system and LODDS. In our study, seven independent prognostic factors, age, marital status, grade, tumor size, AJCC 8^th^ edition T stage, AJCC 8^th^ edition M stage, and LODDS, were selected. Based on these factors, we built a nomogram to evaluate the survival rate for the patients described above.

Through multivariate Cox regression analysis, we found that patients older than sixty-five years may have a relatively poor overall survival. Our results are consistent with those of previous studies showing that elderly patients tend to have higher tumor grades, worse performance statuses, and shorter survival times than younger patients [17, 18]. Similar to the research of Song et al. [19], our study found that marital status actually affected the prognosis of patients with gallbladder adenocarcinoma. Patients who were unmarried or divorced would have relatively poor survival after surgery. Some studies have shown that unmarried patients are more likely to be affected by depression and anxiety from a biological and social psychological point of view because they have no spouse, which in turn affects their willingness to undergo follow-up treatment and their confidence in recovery after receiving treatment [19–21]. As a result, these patients have a worse prognosis than married patients. This theory was confirmed by our research. Tumor grade is closely related to the biological behavior of different kinds of tumors, so it naturally affects the prognosis of patients [1, 22]. In our research, poorly differentiated patients accounted for 37.0%. Our analysis and survival curve showed that when the tumor's pathological grade changed from better to worse, the patient's OS changed subsequently. This result is consistent with the conclusions from previous studies [1, 22]. The AJCC TNM stage is an important indicator for judging the degree of tumor progression, choosing treatment options, and determining prognosis [10, 23]. In our results, the AJCC T stage and AJCC M stage were considered to be independent prognostic factors. Lymph node metastasis is the most important prognostic factor, and many studies show that only adequate lymph node dissection will give patients a better prognosis [24–27]. In our research, we did not use traditional AJCC lymph node staging. Instead, we used a completely new method of lymph node staging: LODDS [27–29]. Unlike traditional lymph node staging, LODDS is a new method of lymph node staging that has been proposed for a long time. It has been well validated in many tumor models and has been shown to play a better role than the latest eighth version of lymph node staging for GBC [30, 31]. Compared with simply classifying lymph nodes based on the presence of lymph node metastasis or the number of lymph node metastases, LODDS can better eliminate the impact of the total number of lymph nodes removed by surgery on the detection rate of positive lymph nodes [28, 29]. From our results, we can see that the degree of lymph node dissection has no practical value in judging the prognosis of patients, and similar results have been obtained in studies on pancreatic cancer [14]. This may be because only the number of total lymph nodes dissected was considered. Many studies have shown that the rate of positive lymph nodes is closely related to the prognosis of patients with malignant tumors, such as GBC [24, 26–29, 32]. The AJCC recommends the removal of at least 12 lymph nodes to be sufficient for accurate tumor staging [10]. The reason is as we mentioned before. X-tile was developed by Yale University and has been proven to provide an accurate cut-off value for index stratification [33]. It has been widely applied in the evaluation index stratification of different diseases, and we used it to stratify tumor size [14, 34]. From our results, a larger tumor size indicated a worse prognosis in different tumors [9, 30]. Compared with tumors smaller than 1.9 cm, tumors larger than 4.8 cm differed by nearly 30% in the five-year survival rate in our survival curve.

In our research, we set up a modeling cohort and an internal validation cohort according to previous studies [13, 14]. The C-index in our training cohort was 0.735 (95% CI, 0.716 to 0.754), which denoted medium prediction accuracy (0.71-0.90). It demonstrated better discrimination and ability to provide patients with personalized predictions. The AIC of our nomogram was 8658. We found that our nomogram based on LODDS had a more accurate evaluation effect than the 8^th^ edition of the AJCC TNM staging system (C-index: 0.693, 95% CI: 0.674-0.712; AIC: 8763). Through calibration curve and ROC curve verification, our model showed the expected consistency for predicting 1-, 3-, and 5-year overall survival. These results mean that our nomogram could accurately judge the prognosis of patients with gallbladder adenocarcinoma resection over 45 years old. Utilizing the survival prognosis nomogram we obtained, gallbladder adenocarcinoma patients whose tumor sizes were both 3 cm were taken as examples (Table 3): A 55-year-old married patient with AJCC TNM stage T2, M0, LODDS2, and pathological grade II scored 78 points, and the 1-, 3-, and 5-year OS rates were 87%, 63%, and 53%; the other patient was 75 years old and unmarried with AJCC TNM stage T2, M0, LODDS2, and pathological grade IV and scored 186 points; the 1-, 3-, and 5-year OS rates were 55%, 15%, and 0%. Through these results, we can see that, unlike the traditional AJCC TNM stage, even if two patients have the same TNM stage, we can obtain different prognosis predictions through our nomogram. Using our nomogram, different evaluation indicators can be accurately calculated by scores, and the score of each indicator can be further added to the total score. Through the total score, we can accurately predict the 1-, 3-, and 5-year OS rates of patients. Our nomogram provides a more accurate prognostic evaluation for patients with gallbladder adenocarcinoma. We are confident that our nomogram can help clinicians to make a good assessment of the prognosis of patients undergoing gallbladder adenocarcinoma resection who are older than 45 years.

Our research has some strengths. First, our nomogram was based on the SEER database, which collected clinical data from 28% of the US population. This means that our model is supported by a large amount of data. Second, compared with previous studies, our model targeted patients with gallbladder adenocarcinoma older than 45 years old, who account for the majority of GBC patients. Finally, calibration curve and ROC curve verification found that our nomogram showed good consistency in the modeling cohort and verification cohort, and the C-index and AIC were better than that of the AJCC 8^th^ TNM stage. This means that our nomogram has a good prognostic effect.

There are also some limitations. First, this was a retrospective study based on the SEER database, so selective bias was inevitable. Second, the establishment of our nomogram has only passed the internal verification of the dataset and lacks external data verification as support. Third, the SEER database cannot obtain the basic indications of patients and the occurrence of complications; thus, the impact of these factors on prognosis could not be evaluated. Future studies need to build a more comprehensive nomogram to provide perfect predictions for patient prognosis.

## 5. Conclusions

Age, marital status, grade, tumor size, AJCC 8^th^ edition T stage, AJCC 8^th^ edition M stage, and LODDS were independent prognostic factors for patients over 45 years old with gallbladder adenocarcinoma. Using these factors, we established a new nomogram as an accurate and convenient method for the assessment of patient prognosis. Our nomogram has a more accurate prognostic evaluation ability than the 8^th^ edition of the AJCC TNM stage.

## Figures and Tables

**Table 1:** Clinical and pathological features of patients with gallbladder adenocarcinoma over 45 years old.

Variable	Variable level	*N*	Training cohort	Validation cohort	*P* value
		(*n* = 1612)	(*n* = 1212)	(*n* = 400)	
		*n* (%)	*n* (%)	*n* (%)	
Age	<65	543 (33.7)	412 (34.0)	131 (32.8)	0.648
	≥65	1069 (66.3)	800 (66.0)	269 (67.2)	
Marital status	Married	895 (55.5)	675 (55.7)	220 (55.0)	0.809
	Unmarried	717 (44.5)	537 (44.3)	180 (45.0)	
Race	White	1213 (75.2)	917 (75.7)	296 (74.0)	0.362
	Black	194 (12.0)	138 (11.4)	56 (14.0)	
	Other	205 (12.7)	157 (13.0)	48 (12.0)	
Sex	Male	492 (30.5)	381 (31.4)	111 (27.8)	0.165
	Female	1120 (69.5)	831 (68.6)	289 (72.2)	
Grade	Well, I	218 (13.5)	166 (13.7)	52 (13.0)	0.692
	Moderately, II	775 (48.1)	580 (47.9)	195 (48.8)	
	Poorly, III	596 (37.0)	451 (37.2)	145 (36.2)	
	Undifferentiated, IV	23 (1.4)	15 (1.2)	8 (2.0)	
AJCC 8^th^ T stage	T1	150 (9.3)	112 (9.2)	38 (9.5)	0.363
	T2	802 (49.8)	604 (49.8)	198 (49.5)	
	T3	602 (37.3)	458 (37.8)	144 (36)	
	T4	58 (3.6)	38 (3.14)	20 (5.0)	
LODDS	LODDS1	518 (32.1)	397 (32.8)	121 (30.2)	0.648
	LODDS2	642 (39.8)	478 (39.4)	164 (41.0)	
	LODDS3	452 (28.0)	337 (27.8)	115 (28.7)	
AJCC 8^th^ M stage	M0	1365 (84.7)	1024 (84.5)	343 (85.8)	0.542
	M1	245 (15.2)	188 (15.5)	57 (14.2)	
LN surgery scope	None	40 (2.5)	31 (2.6)	9 (2.3)	0.942
	1 ~ 3	1080 (67.0)	811 (66.9)	269 (67.2)	
	4~	492 (30.5)	370 (30.5)	122 (30.5)	
Radiation	Yes	385 (23.9)	299 (24.7)	86 (21.5)	0.197
	No/unknown	1227 (76.1)	913 (75.3)	314 (78.5)	
Chemotherapy	Yes	678 (42.1)	513 (42.3)	165 (41.2)	0.705
	No/unknown	934 (57.9)	699 (57.7)	235 (58.8)	
Tumor size (cm)	<1.9	355 (22.0)	273 (22.5)	82 (20.5)	0.218
	1.9~4.8	895 (55.5)	657 (54.2)	238 (59.5)	
	≥4.8	362 (22.5)	282 (23.3)	80 (20.0)	

AJCC: American Joint Committee on Cancer; LODDS: log of positive lymph nodes; LN: lymph nodes.

**Table 2:** Univariate and multivariate Cox regression analyses of OS for patients with gallbladder adenocarcinoma in the training cohort.

Characteristics	Variable level	Univariate analysis			Multivariate analysis		
		HR	95% CI	*P* value	HR	95% CI	*P* value
Age (years)				<0.001^∗∗∗^			<0.001^∗∗∗^
	≤65	Reference			Reference		
	>65	1.55	1.317-1.824	<0.001^∗∗∗^	1.686	1.400-1.988	<0.001^∗∗∗^
Marital status				0.016^∗^			0.001^∗∗^
	Married	Reference			Reference		
	Unmarried	1.2	1.035-1.39	0.016^∗^	1.298	1.117-1.508	0.001^∗∗^
Race				0.003^∗∗^			0.11
	White	Reference					
	Black	0.757	0.591-0.971	0.028^∗^			0.47
	Other	0.712	0.559-0.906	0.006^∗∗^			0.062
Sex				0.516			
	Male	Reference					NI
	Female			0.516			
Grade				<0.001^∗∗∗^			0.06
	I	Reference			Reference		
	II	1.385	1.071-1.79	0.013^∗^	1.234	0.952-1.600	0.112
	III	1.947	1.503-2.523	<0.001^∗∗∗^	1.237	0.947-1.615	0.119
	IV	4.396	2.474-7.81	<0.001^∗∗∗^	2.295	1.282-4.109	0.005^∗∗^
T stage				<0.001^∗∗∗^			<0.001^∗∗∗^
	T1	Reference			Reference		
	T2	1.565	1.112-2.202	0.01^∗^	1.229	0.868-1.741	0.245
	T3	3.639	2.59-5.114	<0.001^∗∗∗^	2.424	1.699-3.459	<0.001^∗∗∗^
	T4	6.902	4.287-11.112	<0.001^∗∗∗^	3.715	2.262-6.101	<0.001^∗∗∗^
LODDS				<0.001^∗∗∗^			<0.001^∗∗∗^
	LODDS1	Reference			Reference		
	LODDS2	1.525	1.262-1.842	<0.001^∗∗∗^	1.316	1.087-1.593	0.005^∗∗^
	LODDS3	3.228	2.659-3.919	<0.001^∗∗∗^	2.515	2.053-3.080	<0.001^∗∗∗^
M stage				<0.001^∗∗∗^			
	M0	Reference			Reference		
	M1	3.04	2.531-3.651	<0.001^∗∗∗^	2.067	1.704-2.508	<0.001^∗∗∗^
LN surgery scope				0.003^∗∗^			
	None	Reference					NI
	1~3	1.462	0.825-2.593	0.193			
	≥4	1.12	0.625-2.005	0.704			
Radiation				0.289			
	Yes	Reference					NI
	No/unknown	1.217	0.991-1.493	0.289			
Chemotherapy				0.512			
	Yes	Reference					NI
	No/unknown	1.084	0.925-1.271	0.512			
Tumor size (cm)				<0.001^∗∗∗^			0.003^∗∗^
	<1.9	Reference			Reference		
	1.9~4.8	1.605	1.31-1.965	<0.001^∗∗∗^	1.176	0.952-1.453	0.132
	≥4.8	2.611	2.084-3.271	<0.001^∗∗∗^	1.503	1.179-1.916	0.001^∗∗^

AJCC: American Joint Committee on Cancer; LODDS: log of positive lymph nodes. Level of significance: ^∗^*P* < 0.05; ^∗∗^*P* < 0.01; ^∗∗∗^*P* < 0.001.

**Table 3:** Comparison of 1-, 3-, and 5-year OS of two gallbladder adenocarcinoma patients with a tumor size of 3 cm according to the nomogram.

Variable	Patient 1			Patient 2		
	Value	Points	1-, 3-, 5-year OS	Value	Points	1-, 3-, 5-year OS
Age	55	0		75	40	
Marital status	Married	0		Unmarried	18	
Grade	II	15		IV	65	
AJCC T stage	T2	14		T2	14	
AJCC M stage	M0	0		M0	0	
LODDS	LODDS2	38		LODDS2	38	
Tumor size (cm)	3	11		3	11	
Total points		78	87%, 63%, 53%		186	55%, 15%, 0%

AJCC: American Joint Committee on Cancer; LODDS: log of positive lymph nodes.

## Data Availability

The data used to support the findings of this study are included within the article.

## Abbreviations

GBC: Gallbladder cancer
LODDS: Log of positive lymph nodes
AJCC: American Joint Committee on Cancer
OS: Overall survival
SEER: Surveillance, Epidemiology, and End Results
C-index: Concordance index
AUC: Area under the curve
ROC: Receiver operating characteristic.

## Supplementary Materials

Supplementary Materials Figure S1: X-tile plots identifying the cut-off of Tumor Size (A, B, C), LODDS (D, E, F). Abbreviations: 16 LODDS: Log odds of positive lymph nodes. Table S1: process of data screening in the SEER database.
