# Supplementary material for: Prognostic Evaluation for Patients over 45 Years Old with Gallbladder Adenocarcinoma Resection: A SEER-Based Nomogram Analysis
Source: Biomed Res Int. 2020 Jul 18;2020:6370946. doi: 10.1155/2020/6370946 (PMC7383319; doi:10.1155/2020/6370946)
Supplement: Supplementary Materials — Figure S1: X-tile plots identifying the cut-off of Tumor Size (A, B, C), LODDS (D, E, F). Abbreviations: 16 LODDS: Log odds of positive lymph nodes. Table S1: process of data screening in the SEER database. [file 6370946.f1.zip › 6370946.f1/TableS1.docx]

| **Inclusion criteria and exclusion criteria** | **Program selection codes** |
| --- | --- |
| Included cases of gallbladder cancer diagnosed from 2004 to 2016 | Included {Year of diagnosis} = ‘2004’, ‘2005’, ‘2006’, ‘2007, ‘2008’, ‘2009’, ‘2010’, ‘2011’, ‘2012’, ‘2013’, ‘2014’, ‘2015’, ‘2016’ AND {Site recode ICD-O-3/WHO 2008} = ‘Gallbladder’ |
| Included patients with gallbladder adenocarcinoma | Included {Histologic Type ICD-O-3} = ‘8140’ |
| Included patients with a pathological diagnosis | Included {Diagnostic Confirmation} = ‘pathological diagnosis’ |
| Included patients with age ≥45 | Included {Age at diagnose} = ‘>=45’ |
| Excluded patients with unknown marital status at diagnosis | Excluded {Marital status at diagnosis} = ‘Unknown’ |
| Excluded patients with unknown race | Excluded {Race recode (White, Black, Other)} = ‘Unknown’ |
| Excluded patients with unknown grade | Excluded {Grade} = ‘unknown’ |
| Excluded patients with unknown AJCC T stage | Excluded {Derived AJCC T Group, 7th ed (2010-2015)} = ‘NA’, ‘T0’, ‘T1NOS’, ‘TX’;{Derived AJCC T Group, 6th ed (2004-2015)}= ‘NA’, ‘T0’, ‘T1NOS’, ‘TX’; {Derived SEER Combined T (2016+)}= ‘cX’, ‘pX’, ‘Not applicable’ |
| Excluded patients with unknown AJCC M stage | Excluded {Derived AJCC M Group, 7th ed (2010-2015)} = ‘NA’; {Derived AJCC Stage Group, 6th ed (2004-2015)} = ‘NA’, ‘T0’, ‘T1NOS’, ‘TX’; {Derived SEER Combined N (2016+)} = ‘cX’, ‘pX’, ‘Not applicable’ |
| Excluded patients with unknown surgical approach | Excluded {RX Summ--Surg Prim Site (1998+)} = ‘0’, ‘99’ |
| Excluded patients with unknown regional nodes examined | Excluded {Regional nodes examined (1988+)} = ‘95’, ‘96’, ‘97’, ‘98’, ‘99’ |
| Excluded patients with unknown regional nodes positive | Excluded {Regional nodes positive (1988+)} = ‘95’, ‘96’, ‘97’, ‘98’, ‘99’ |
| Included patients with clear Scope Reg LN Sur | Included {RX Summ--Scope Reg LN Sur (2003+)} = ‘None’, ‘1 to 3 regional lymph nodes removed’, ‘4 or more regional lymph nodes removed’ |
| Excluded patients with unknown tumor size | Excluded {CS tumor size (2004-2015)} = ‘990’, ‘999’ |
