# Supplementary figures and images for: Prognostic Evaluation for Patients over 45 Years Old with Gallbladder Adenocarcinoma Resection: A SEER-Based Nomogram Analysis
Source: Biomed Res Int. 2020 Jul 18;2020:6370946. doi: 10.1155/2020/6370946 (PMC7383319; doi:10.1155/2020/6370946)

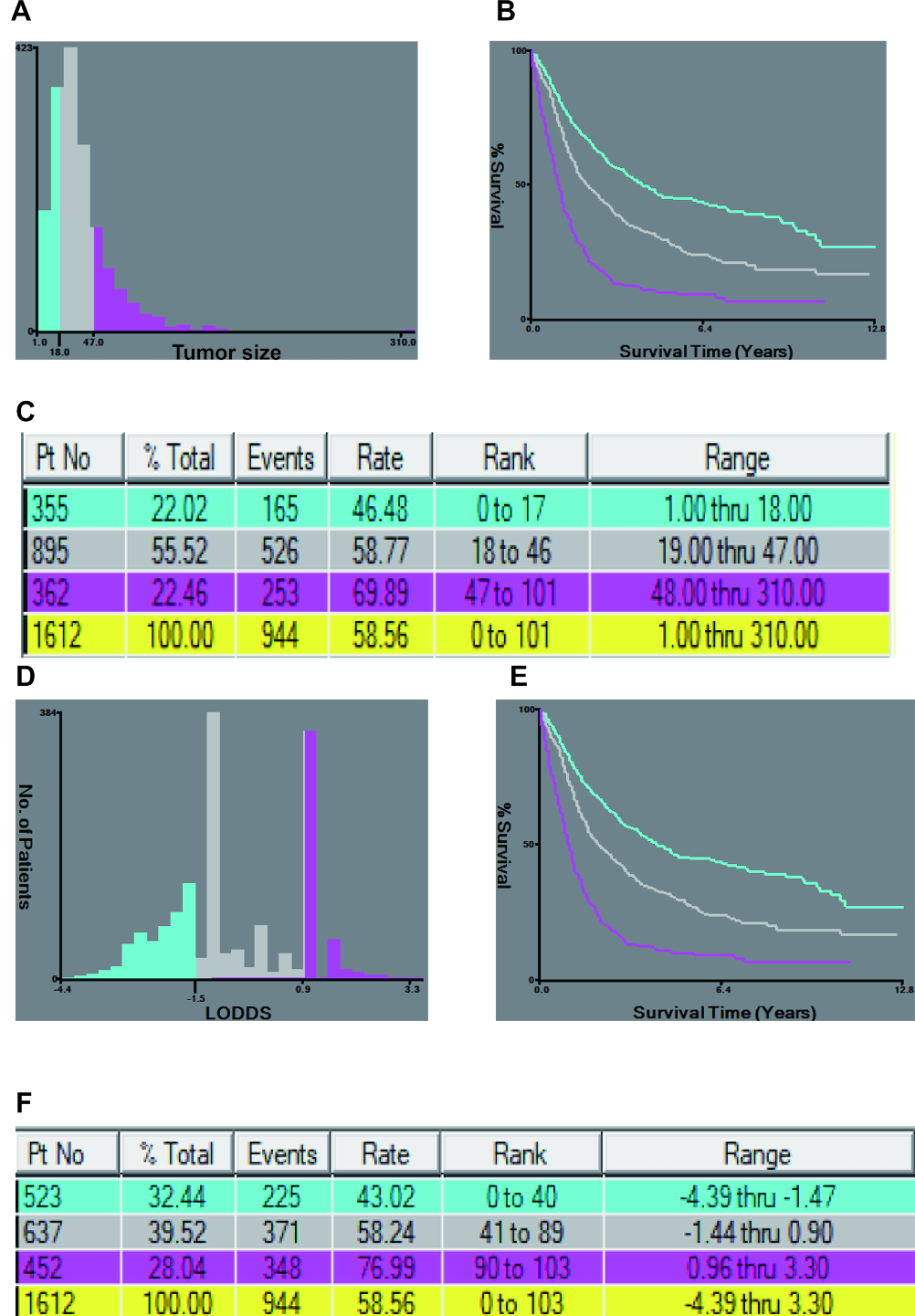

Supplement: Supplementary Materials — Figure S1: X-tile plots identifying the cut-off of Tumor Size (A, B, C), LODDS (D, E, F). Abbreviations: 16 LODDS: Log odds of positive lymph nodes. Table S1: process of data screening in the SEER database. [file 6370946.f1.zip › 6370946.f1/FigS1.tif]
